# The protective effect of growth hormone on Cu/Zn superoxide dismutase-mutant motor neurons

**DOI:** 10.1186/s12868-015-0140-z

**Published:** 2015-02-06

**Authors:** Jin-Young Chung, Hyun-Jung Kim, Manho Kim

**Affiliations:** Department of Veterinary Internal Medicine and Geriatrics, Kangwon National University, Gangwondo, South Korea; Department of Neurology, Seoul National University Hospital, 101 Daehakro, Chongno-ku, 110-744 Seoul, South Korea; Protein Metabolism Medical Research Center, College of Medicine, Seoul National University, 101 Daehakro, Chongno-ku, 110-744 Seoul, South Korea

**Keywords:** Amyotrophic lateral sclerosis (ALS), Growth hormone (GH), Mutated SOD1

## Abstract

**Background:**

Amyotrophic lateral sclerosis (ALS) is characterized by selective degeneration of motor neurons. The gene encoding Cu/Zn superoxide dismutase (SOD1) is responsible for 20% of familial ALS cases. Growth hormone (GH) concentrations are low in the cerebrospinal fluid of patients with ALS; however, its association with motoneuronal death is not known. We tested the neuroprotective effects of GH on human SOD-1-expressing cultured motor neurons and SOD1G93A transgenic mice.

**Results:**

In cultured motor neurons, cytotoxicity was induced by A23187, GNSO, or homocysteine, and the effects of GH were determined by MTT, bax, PARP cleavage pattern, Hoechst nuclear staining, MAPK, and PI3K assay. In SOD-1 transgenic mice, rotarod motor performance was evaluated. Survival analysis of motoneuronal loss was done using cresyl violet, GFAP, and Bcl-2 staining. GH prevents motorneuronal death caused by GSNO and homocysteine, but not that by A23187. It activates MAPK and PI3K. GH-treated mice showed prolonged survival with improved motor performance and weight loss. GH decreased cresyl violet positive motoneuronal loss with strong Bcl-2 and less GFAP immunoreactivity.

**Conclusions:**

Our results demonstrate that GH has a protective effect on mutant SOD-1-expressing motor neurons.

## Background

Amyotrophic lateral sclerosis (ALS) is a degenerative disorder [1,2] characterized by selective degeneration of motor neurons predominantly in the anterior horn of the spinal cord and brainstem, and pyramidal cells of the motor cortex. ALS leads to progressive weakness, atrophy of skeletal muscles, and eventual paralysis and death, usually within 2–5 years [3].

Up to 10% of ALS cases are familial (fALS); the remaining 90% have no hereditary component and are known as sporadic ALS [4]. About 20% of fALS cases are caused by autosomal dominant mutations in the gene encoding for Cu/Zn superoxide dismutase (SOD1) [5]. Pathophysiological mechanisms of fALS due to SOD1 mutations include a failure to fold or degrade mutant SOD1, production of free radicals, release of free copper, or susceptibility of mutant SOD1 to disulfide reduction [6-8].

Many studies have implicated oxidative stress and an excitotoxic mechanism in the pathogenesis of ALS. Plasma homocysteine (HC), which is continuously produced from diet and increases with age, has been considered to play a role in motor neuronal death, thus resulting in ALS. HC in vascular systems produces reactive oxygen species (ROS), such as superoxide anions, and reduces transitional metals by reaction of the metal with molecular oxygen. NO (Nitric oxide) might be involved in the mechanism of action of HC. NO is scavenged for transformation into peroxynitrite by reaction with the superoxide anion generated by HC. This reactive peroxynitrite causes cell death by oxidative damage, disruption of energy metabolism, calcium homeostasis, and mitochondrial function [9,10]. In addition, motor neurons in SOD1 models are vulnerable to excitotoxicity by glutamate; glutamate receptor-mediated neurodegeneration is associated with calcium influx and substantial intracellular calcium accumulation [11,12].

Growth hormone (GH), a high molecular weight peptide composed of 191 amino acids, is produced by the anterior lobe of the pituitary gland. GH functions either by direct action on tissues or through the insulin-like growth factor-1. It causes proliferation of many types of cells and controls differentiation in adipose or muscle tissues. It also controls the metabolism of proteins, carbohydrates, or fatty acids [13,14]. Although the effect of GH on the central nervous system (CNS) was first reported over 60 years ago, virtual data have appeared in the past decade [15-17]. GH secretion was studied in myotonic dystrophy. This condition was associated with an abnormal pattern of GH secretion over 24 hours and a significant decrease in its circulating levels [18,19]. One study reported a reduction of GH secretion in patients with ALS [20].

In the present study, we examined the protective effect of GH on motor neuronal death. We tested the effect of GH on the viability of motoneuron-neuroblastoma hybrid cells (VSC4.1) expressing mutated human SOD1 against cytotoxic stimuli. In addition, we attempted to determine whether GH treatment has a neuroprotective effect on transgenic mice with the SOD1G93A mutation. Lifespan and body weight measurements, rotarod test, and immunohistochemistry for motor neuronal loss were performed.

## Results

### The effect of GH on mutant human SOD1 cell lines

Cell lines were cultured with various GH concentrations for 24 h (0.0004 I.U./ml to 0.4 I.U./ml). The number of living cells increased in the A4V cell line (p = 0.015) and G93A cell line (p = 0.034) as determined by the MTT assay. However, there was no statistical difference with the number of viable cells in the WT cell line.

**Figure 1:**
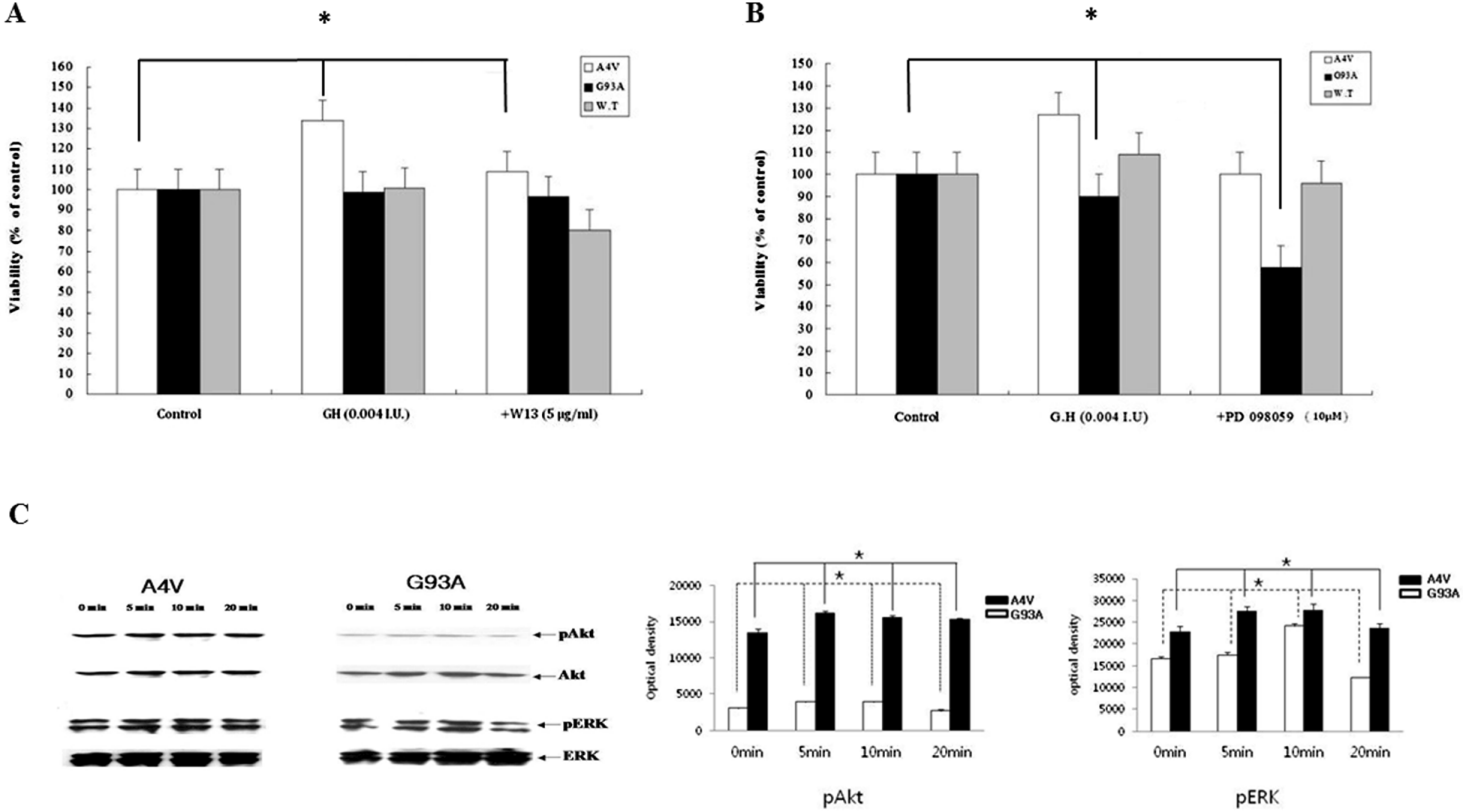
**PI3-kinase or MAP-kinase inhibitor treatment and activation of pAkt and pERK by GH.** Increased viability of A4V was reduced by W13 (p = 0.039) **(A)**. Increased viability of G93A was reduced by treatment with PD098059, a MAP-kinase inhibitor (p = 0.038) **(B)**. GH was applied at 0.004 I.U/ml and active pAkt and pERK forms were identified 5, 10, and 20 min later. The change in levels of pAkt and pERK in A4V and G93A cell lines at different time points were analyzed **(C)**. Results are expressed as mean values and SD (*p < 0.05).

When treated with W13 (P13K inhibitor), the increasing viability of A4V with pretreated GH decreased (p = 0.039) (Figure 1A). However, it was not significantly different from the WT and G93A cell lines. When treated with P098050 (MAPK inhibitor), the increasing viability of G93A with pretreated GH decreased (p = 0.038) (Figure 1B). However, the difference was not significant from that of the WT and A4V cell lines. For immunoblotting studies, A4V and G93A cell lines were treated with GH (0.004 I.U./ml) and active pAkt and pERK forms were identified. The change of pAkt in A4V cell line (p = 0.022) and G93A cell line (p = 0.034) at different time points had statistical significance. Similarly, pERK levels also changed significantly in A4V (p = 0.038) and G93A cell lines (p = 0.019) at different time points. In A4V cells, the ratio of pAkt and pERK were higher than those in G93A but the difference was not significant (p = 0.05) (Figure 1C).

### The protective effect of GH on cytotoxicity

In previous studies, treatment with A23187, calcium influx, GSNO, NO-donor, and homocysteine had a selective cytotoxic effect in the mutated SOD-1 cell lines, G93A, and A4V. Viabilities of G93A and A4V decreased compared to that of WT [9,21]. In this study, these effects were reconfirmed.

#### Lack of the protective effect of GH on cytotoxicity of A23187

When treated with A23187 (5 μM), cell viabilities decreased but did not reach significance. Viabilities in A4V and G93A decreased by 10% and 20%, respectively, which was higher than that in WT. Pretreatment with 0.04 I.U./ml and 0.4 I.U./ml GH had no protective effect on WT, A4V, and G93A cell lines.

#### The protective effect of GH on the cytotoxicity of GSNO

**Figure 2:**
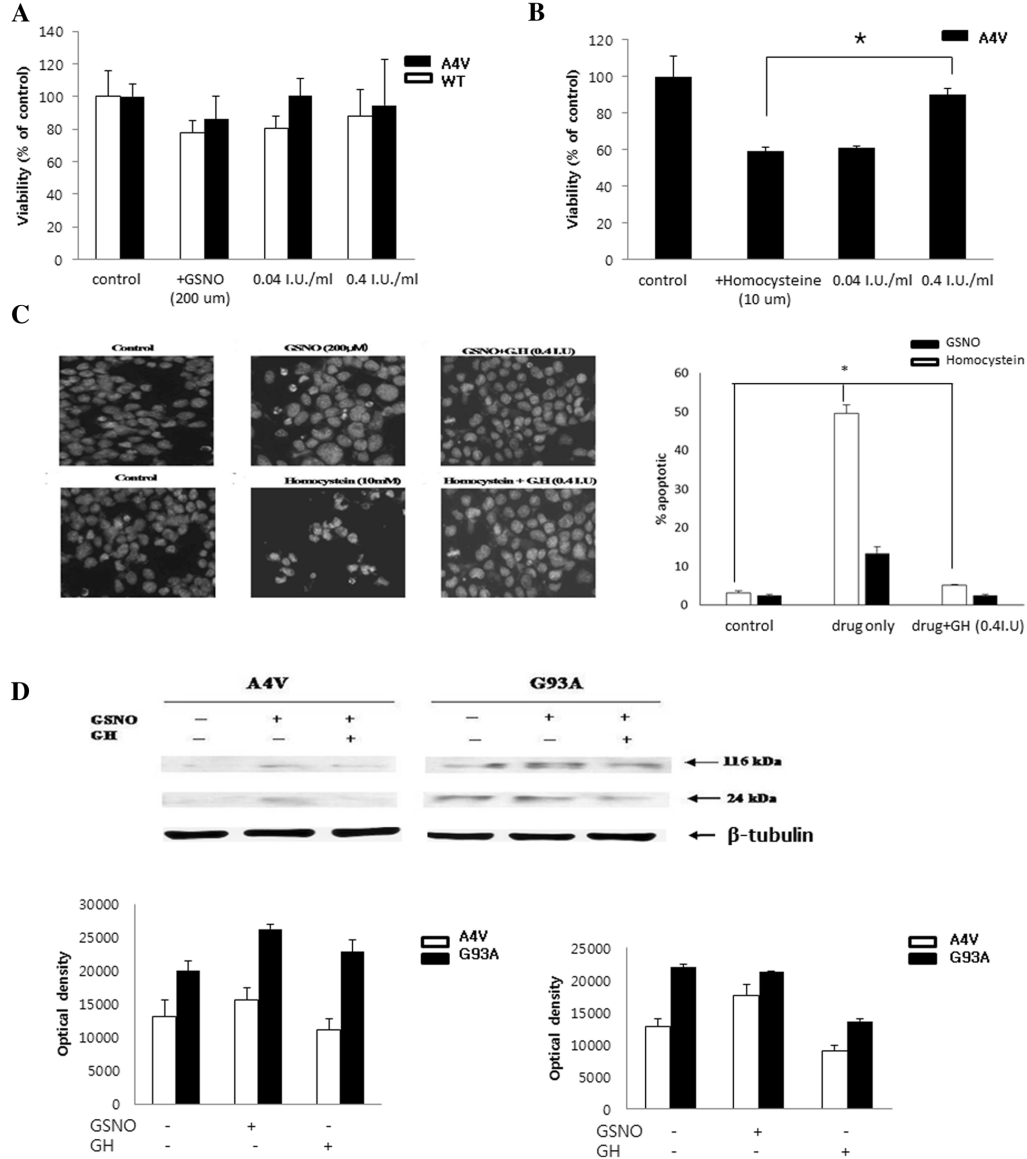
**Cytoprotective effect of GH.** The protective effect of GH with GSNO pretreatment was observed in mutant cells **(A)**. In homocysteine-treated cells, reduced viabilities in A4V were partly reversed by GH at 0.4 I.U/ml (p = 0.046) **(B)**. GSNO treatment increased nuclear fragmentation; GH treatment reduced this phenomenon. Homocysteine also reduced cell viability and the number of fragmented nuclei; this was reversed by pretreatment with GH (p = 0.039) **(C)**. GSNO treatment increased 116 kD and 24 kD PARP fragments in A4V and G93A cells, which were reduced by GH treatment **(D)**. Results are expressed as mean values and SD (*p < 0.05).

GSNO (200 μM) treatment caused a non-significant reduction in viabilities. The reduction was 20% in A4V and 10% in G93A, and was higher than in WT. Pretreatment with 0.04 I.U./ml and 0.4 I.U./ml GH caused viabilities in WT and A4V to increase, but not significantly (Figure 2A). Nuclear fragmentation was observed after GSNO (200 μM) treatment which decreased by pretreatment with 0.4 I.U./ml GH, but there were no statistically significant difference (Figure 2C). While there were no changes in Bax expression, PARP expression (24 kDa, 116 kDa) increased after treatment with GSNO 200 μM, but protective effect of GH was not observed in A4V and G93A cell lines (Figure 2D).

#### The protective effect of GH on the cytotoxic effect with Homocysteine

When treated with homocysteine (10 μM), cell viabilities decreased but was not significantly different compared to WT. Viabilities in A4V and G93A decreased more than that in WT (by 20% and 40%, respectively). The protective effect of GH was observed in A4V, especially at 0.4 I.U./ml (p = 0.046) (Figure 2B). Homocysteine also caused nuclear fragmentation which decreased by pretreatment with 0.4 I.U./ml GH (p = 0.039) (Figure 2C); however, there were no changes in Bax and PARP expression.

### GH improves motor performance

**Figure 3:**
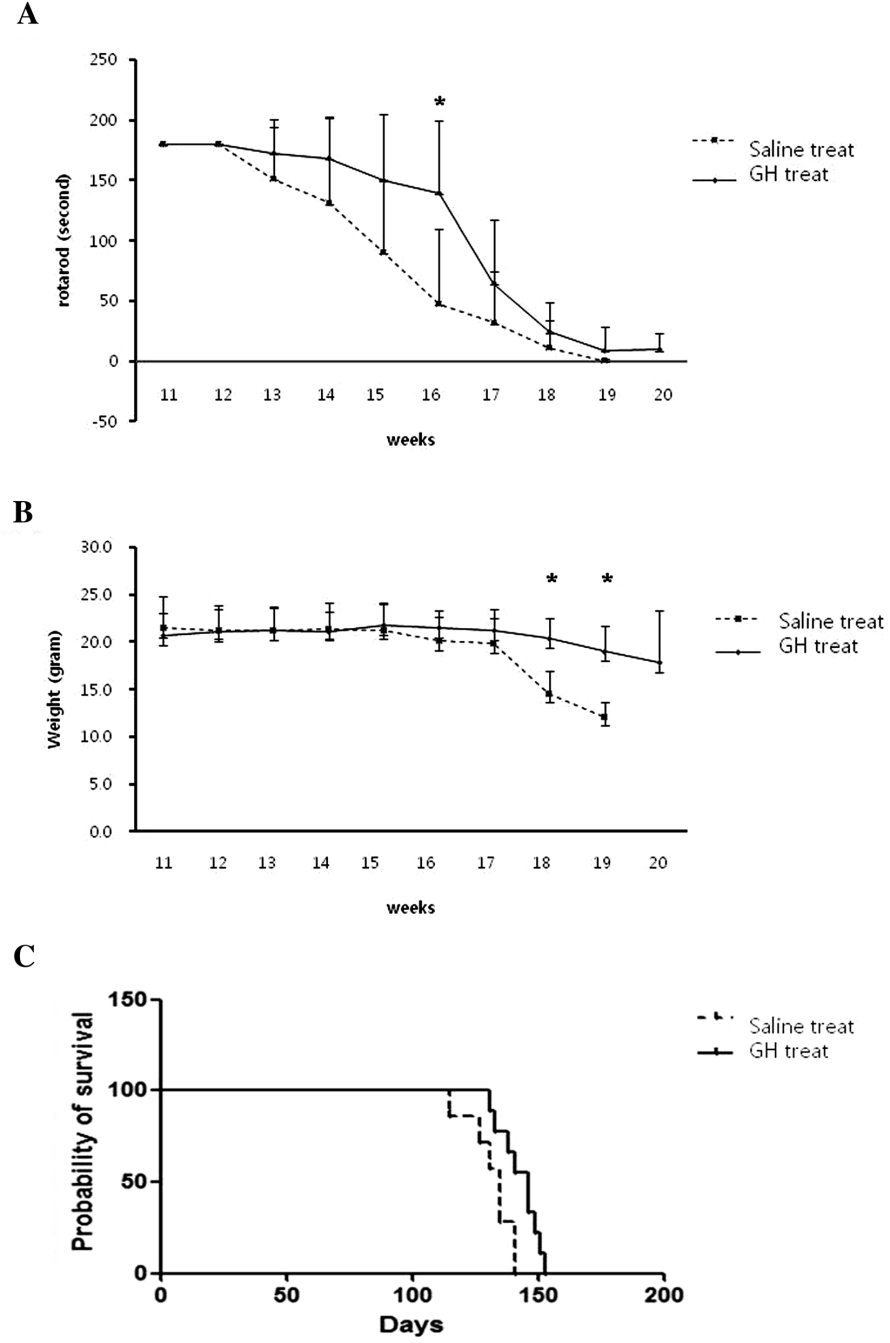
***In vivo*** **study.** In the rotarod test, GH treatment significantly improved motor performance at 16 weeks, compared to saline-treated SOD1G93A mice (p = 0.017) **(A)**. GH treatment significantly improved weight loss at 18 (p = 0.004) and 19 weeks (p = 0.040) **(B)**. GH treatment resulted in a significant improvement in lifespan, compared with saline-treated SOD1G93A transgenic mice (p = 0.019) **(C)**. Y error bars represent standard error of the mean (±SD) (*p < 0.05).

To investigate improvements in balance and coordination, we used the rotarod test. GH treatment significantly improved motor performance at 16 weeks, compared to saline-treated SOD1G93A mice (P = 0.017) (Figure 3A).

### GH improves weight loss

In order to examine weight loss, we monitored mouse body weights weekly. Mice in both groups were weighed from 11 weeks of age. GH treatment significantly improved weight loss at 18 weeks (p = 0.004) and 19 weeks (p = 0.040) (Figure 3B).

### GH improves lifespan

To determine the protective effects of GH on lifespan, GH was injected weekly from postnatal 11 weeks till death (5.583 μg/kg, i.p.) in GH-treated SOD1G93A transgenic mice. After treatment, lifespan was compared with saline-treated SOD1G93A transgenic mice. GH-injection resulted in a significant improvement in lifespan (P = 0.019) (Figure 3C). Mean mouse survival increased from 132 ± 9 days to 143 ± 8 days.

### Immunoreactivity analysis of the effect of GH on motor neuron loss

**Figure 4:**
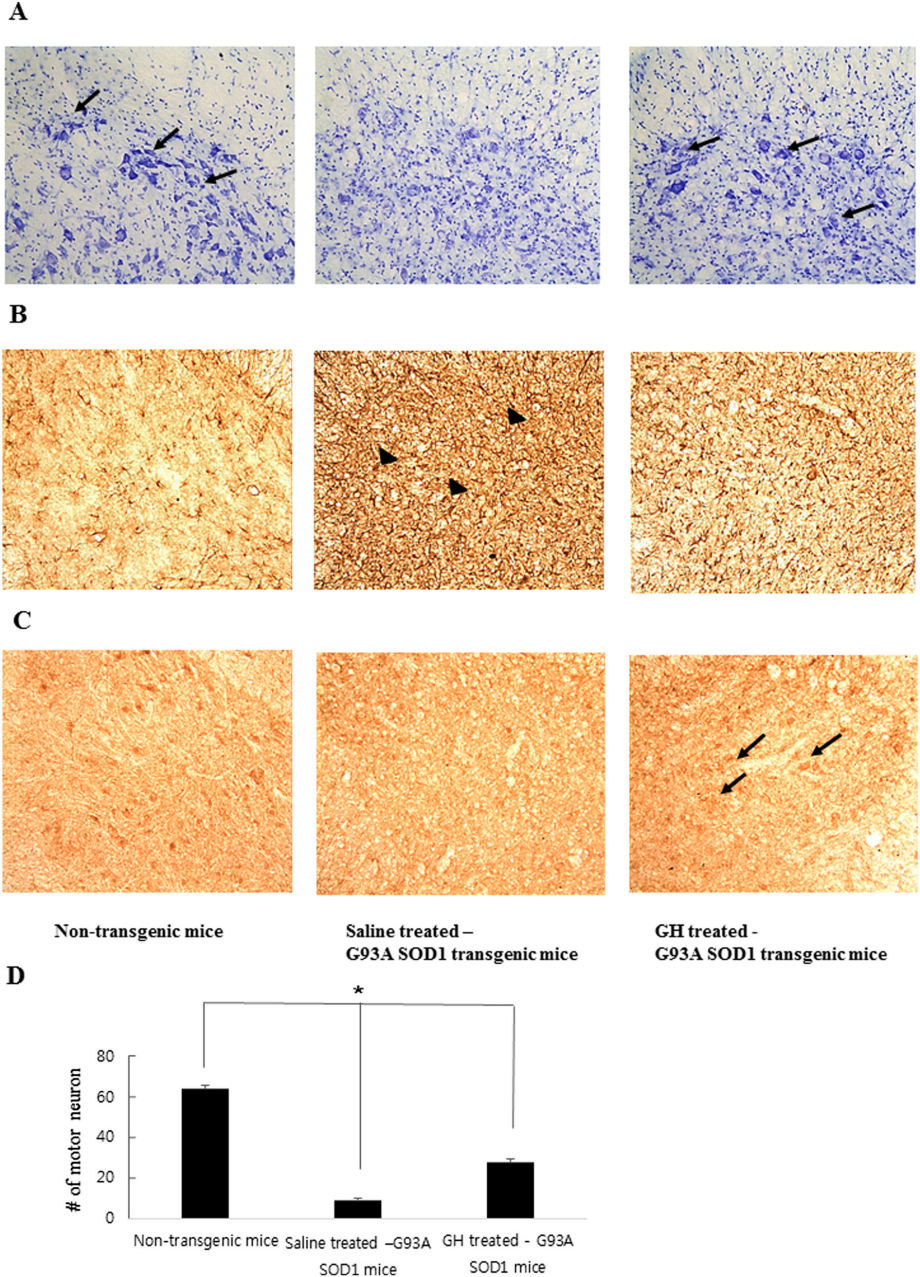
**Histopathological analysis by cresyl violet, GFAP, and Bcl-2 staining.** Cresyl violet staining revealed few numbers of neurons in saline-treated SOD1G93A mice, and higher numbers in non-transgenic and GH-treated SOD1G93A mice **(A)**. GFAP immunoreactivity was observed at very low levels in non-transgenic mice, and at significantly higher levels in saline-treated SOD1G93A mice **(B)**. Increasing GFAP immunoreactivities in saline-treated SOD1G93A mice were attenuated by GH treatment. Bcl-2 staining **(C)** is stronger in GH-treated SOD1G93A mice than in saline-treated SOD1G93A mice. (×200) The number of motor neurons is significantly higher in the non-transgenic and GH-treated SOD1G93A mice (p = 0.027) **(D)**. Results are expressed as mean values and SD (*p < 0.05).

In GH-treated SOD1G93A transgenic mice, there was a reduction in the number of cresyl violet-positive neurons, compared to non-transgenic mice, but a significant increase was noted compared to that in saline-treated SOD1G93A transgenic mice (p = 0.027) (Figure 4A, D). To determine whether GH exerted a neuroprotective effect in SOD1G93A transgenic mice, we examined GFAP expression in astrocytes by immunohistochemistry. Lumbar spinal cord sections of saline-treated SOD1G93A transgenic mice showed strong GFAP immunoreactivity. In contrast, the GFAP signal was attenuated in the lumbar spinal cord of GH-treated SOD1G93A mice and non-transgenic mice (Figure 4B). Next, we performed immunostaining for Bcl-2 in order to determine if GH inhibits apoptotic neuronal death in SOD1G93A transgenic mice, (Figure 4C) and found stronger immunoreactivity compared to saline-treated SOD1G93A mice.

## Discussion

A few studies on halting disease progression in SOD1-dependent ALS have been carried out. The effect of GH on the CNS appears to involve brain growth and development, and action as a neuroprotective factor [15-17]. We tested the neuroprotective effects of GH on human SOD-1-expressing cultured motor neurons and SOD1G93A transgenic mice. The results showed that GH protects against motorneuronal death caused by GSNO and homocysteine, but not by A23187. GH activated MAPK and PI3K. Prolongation of survival was observed in GH-treated mice, along with improved motor performance and weight loss. GH decreased cresyl violet positive motoneuronal loss with stronger Bcl-2 and lesser GFAP immunoreactivity. Our results suggest that GH has a protective effect on mutant SOD-1 expressing motor neuronal death.

Zhang *et al*. [21] demonstrated that folic acid protects motor neurons against increased homocysteine, inflammation, and apoptosis in SOD1G94A transgenic mice. Folic acid, which was applied to SOD1G94A transgenic mice, is regarded as an important factor in homocysteine metabolism. The study showed that folic acid treatment significantly delayed disease onset and prolonged lifespan, accompanied by significantly attenuated plasma homocysteine levels, suppressed activation of microglia and astrocytes, and inhibited expression of inducible nitric oxide synthase (iNOS) and tumor necrosis factor-alpha in the spinal cord. Pyruvate has been known as an anti-oxidant and an energy source. When pyruvate was applied in SOD1G94A transgenic mice, lifespan increased, disease progression slowed, and motor performance improved. However, disease onset was not observed to be delayed in this study [22].

GH levels decrease after peaking during adolescence; one third of people over 65 years of age are deficient in GH. Therefore, GH replacement therapy is used as one of the clinical practices for reducing the aging phenomenon [23]. Despite the direct application of GH in slowing aging, the mechanism underlying cytoprotection afforded by GH remains unknown. Only a few studies have shown that GH administration prevents organ and tissue deterioration due to aging. As a possible mechanism of its action, GH has been suggested to act on antioxidant enzymes in the modulation of regulatory pathways in reduction of ROS generation. Moreover, GH has been shown to prevent age-induced reduction in expression of some components, including cytochrome b and c of the mitochondrial respiratory chain [24,25]. Therefore, we hypothesized that GH has a cytoprotective effect associated with mitochondria in the SOD-1 mutant. Studies on the CNS have shown that GH has a neuroprotective effect against ischemic brain injury, can attenuate trauma-induced depression of spinal cord evoked potentials, and can ameliorate motor dysfunction resulting from spinal cord injury [26-28]. CNS weight increases in overexpression of GH and so does the size of lumbar spinal motor neurons. However, there is no relative evidence to demonstrate a lack of alteration in motoneuron numbers in the CNS of GH receptor-deficient mice. Some studies have shown that GH, Insulin-like Growth Factor-1, and insulin concentrations in cerebrospinal fluid (CSF) are significantly lower in patients with ALS in comparison with the control group [29-33]. However, it is unknown whether GH affects the survival of motor neurons in ALS. Morselli *et al.* [20] suggested that two-thirds of patients with ALS present with growth hormone (GH) deficiency. Based on this data, several clinical trials were performed with GH on patients with ALS [34,35]. However these studies failed to show any significant impact on motor symptoms or patient survival, except in a few patients who seemed to be good GH-responders. Therefore, more studies are needed to investigate the effect of GH on motor neurons.

In our study, GH was protective against GSNO and homocysteine in mutant SOD-1-expressing cells [9,36,37]. However, this effect was not observed in cells treated with A23187, suggesting that GH is not related to calcium-influx-mediated cell death. Additionally, the protective effect of GH appears to be related to nuclear fragmentation and not the Bax protein, an indicator of mitochondria-related cell death. PARP cleavage and caspase 3 represent the signals of apoptosis, which are related to mitochondria-related cell death. Reactive astrocytes in the CNS of SOD1G93A transgenic mice have been shown to express cleaved PARP [21,38-40]. With GSNO treatment, GH decreases PARP cleavage, suggesting that GH may affect caspase 3. However, with homocysteine treatment, GH does not affect PARP cleavage, suggesting that it is not related to caspase 3. Therefore, the protective effect of GH in our study may be attributed to a non-mitochondrial mediated apoptosis, i.e., another non-developmental pathway.

In case of Figure 1A, we showed the proliferating effect of GH on A4V that is blocked by W13. However, in Figure 2A, we attempted the NO cytotoxicity and checked whether this cytotoxicity can be protected by GH. We assumed that Figure 1A could show the significant difference the viability of A4V cells because W13 is directly related with the GH receptor cascade. Figure 2A showed the tendency of increasing viability of A4V cells. The main signaling pathway of GH is through the GH receptor related to the tyrosine kinase Janus tyrosine kinase-2 (JAK2). JAK2 is activated when a GH molecule binds to a dimer of the GH receptor and JAK2, which promotes the phosphorylation of both JAK2 and the GH receptor. Subsequently, STAT 1, 3, and 5 dimerize and translocate to the nucleus in order to activate transcription of the target gene. JAK2 also phosphorylates and potentiates the mitogen-activated protein kinase (MAP kinase) and phosphatidyl inositol-3 kinase (PI3 kinase) cascades to proliferate survival related cells [41]. The proliferative effect of GH was observed at concentrations between 0.0004–0.004 I.U., which was negated by the inhibitors of PI3 kinase or MAP kinase, suggesting that this proliferation is mediated by survival signaling. The effect of PI3K or MAPK appeared to differ among the cell lines. The PI3 kinase inhibitor mainly affected A4V, but not WT and G93A. However, the MAP kinase inhibitor mainly affected G93A, and not WT and A4V. pAkt levels suggested that PI3K signaling [42] is activated in A4V and G93A cell lines. Increased pERK levels also suggested that the MAPK signaling pathway is active in A4V and G93A cell lines.

It would be ideal to use mice of the same sex to avoid possible gender differences. Unintentionally, random assignment results in different sex ratio. However, no behavioral or phenotypic gender difference related to G93A mutation was found in G53A C57BL/6 J transgenic mice [43]. A significant reduction in astrogliosis, as assessed by glial fibrillary acidic protein (GFAP) staining was observed, which suggests delayed astrocyte activation in GH-treated SOD1G93A transgenic mice. Reduced expression of Bcl-2 has been observed in the spinal cord of transgenic mice expressing SOD1 with the G93A mutation [44] and in human patients having ALS without SOD1 mutations [45]. Bcl-2 is an important inhibitor of most types of apoptotic cell death, and may reduce motor neuron losses by obstructing the activation of apoptosis induced by Cu/Zn-SOD mutation [7]. In addition, it has been demonstrated that Bcl-2 expression is reduced in symptomatic SOD1G93A transgenic mice [44].

## Conclusion

Our results demonstrate that GH has a protective effect in mutant SOD-1-expressing motor neuronal death. We confirmed these results with *in vitro* and *in vivo* studies in mutant SOD1 cells and mice, respectively. Our results further indicate that the protection afforded by GH to mutant SOD-1 expressing motor neurons could be through a novel non-mitochondrial mediated pathway that is different from the established pathway. However, more trials are needed in order to further clarify the neuroprotective mechanism of GH action.

## Methods

### Cell culture

Motoneuron-neuroblastoma hybrid cells (ventral spinal cord 4.1 [VSC 4.1]; a generous gift from Dr. SH Appel, Baylor College of Medicine, USA) [14,46], which are a fusion product between neuroblastoma N18TG cells and dissociated embryonic rat ventral spinal cord, were maintained in Dulbecco’s modified Eagles’ medium/F-12 growth medium (Gibco, Grand Island, NY) with Sato’s components (Sigma, St. Louis, MD) and 2% heat-inactivated newborn calf serum (HyClone, Logan, UT). This cell line has certain similarities with mammalian motoneurons such as dibutyryl cAMP- or 8-bromocAMP-inducible choline acetyltransferase, neuron-specific enolase, immunoreactive 200-kDa neurofilament protein, synaptophysin, active voltage-gated calcium channels, and expression of calcium binding proteins (calbindin-D28K and parvalbumin) modulated during differentiation [14,47]. They were grown in log-phase on poly-(l-ornithine)-precoated culture dishes (Falcon, Franklin lakes, NJ) [48,49]. Cells were plated in 96-well plates at a density of 1 × 10^4^ cells per well. For the immunoblotting and SOD activity assay, some cells were seeded in 100 mm dishes at 1 × 10^5^ cells.

### Constructs and establishment of stable cell line

Human SOD-1 cDNA from normal and mutant cells was cloned into the BamH1 and EchoRI1 sites of pcDNA 3.0 (Invitrogen, Carlsbad, CA). Cells were designated as wild-type, A4V (mutant cells having SOD1 with alanine substituted to valine at position 4), and G93A (mutant cells having SOD1 with glycine substituted to alanine at position 93). These constructs were a gift from Dr. Lawrence J. Hay-ward (University of Massachusetts, Boston, USA) [14,46]. Following transfection (Superfect, Qiagen, Valencia, CA), the cells were maintained in a medium that contained G418 at a concentration of 400 μg/ml (Gibco, Grand Island, NY). Single or pooled colonies were used for the experiment after confirming expression of human SOD1 (WT, A4V, G93A) by Western blot analysis using an anti-human SOD1 polyclonal antibody (Calbiochem, La Jolla, CA). These cell lines were grown and differentiated under the same conditions as those of VSC 4.1 cells [48].

### Growth hormone application

Growtrophin 4 I.U./vial (Donga pharmaceutical, Korea) is a highly purified human growth hormone (hGH) produced by the *E.coli* strain K-12 (W3110) which overexpresses the hGH expression vector (pHGH401). The hGH gene is modified to remove a methionyl group. Its physiological potency is equivalent to that of the pituitary-derived hGH. Growtrophin 4 I.U. (1.34 mg)/vial was used and reconstructed with 1 ml distilled water. For the experiment, reconstructed Growtrophin was diluted with culture media. The concentration started from 0.0004 I.U./ml (0.134 μg/ml) to 4.0 I.U./ml (1.34 mg/ml) [14].

### Cell viability assay

The 3-[4,5-dimethylthiazol-2-yl]-2,5-diphenyltertazolium bromide (MTT) assay was used for determination of cell viability. In brief, MTT solution (5 mg/ml in PBS) was added to cultures. Following a 3-h incubation period, the MTT solution was removed and formazan precipitate was dissolved in 200 μl dimethyl sulfoxide (DMSO).

### Hoechst nuclear staining

For staining, cells were plated on coverslips coated with poly-d-lysine (10 μg/ml; Sigma, St. Louis, MD) and were fixed with 4% paraformaldehyde for 20 min. Hoechst 33, 342 dye (molecular probe, Eugene, OR) was diluted at 1: 1000 with phosphate buffered saline (PBS) and incubated for 5 min at room temperature. Morphological changes in the nuclei were observed using a fluorescence microscope. To determine any evidence of apoptosis, nuclear fragmentation or chromatin condensation were examined [50].

### Cytotoxic stimuli

In order to induce cytotoxicity by production of reactive oxygen species (ROS), we used homocysteine and S-nitrosoglutathione (GSNO), a nitric oxide donor, which have been previously reported. For calcium induced cytotoxicity, the ionophore A23187 was used for calcium-influx. The concentrations were used as previously described: GSNO 200 μM, homocysteine 10 mM, A23187 5 μM [9,36]. We evaluated cell viability or cytotoxicity by the MTT assay and Hoechst nuclear staining. Cell viability between WT and mutated SOD1-expressing cells was compared to test the protective effect of GH in mutant SOD1 cells; cells were preincubated with GH for 30 min before the cytotoxic stimuli.

### PI3K (Phosphatidylinositol 3-kinase) and MAPK (Mitogen Activated Protein Kinase) assay

PI3K and MAPK were inhibited using wortmannin (W13: Sigma, St. Louis, MO), and 10 μM P098050 (Sigma, St. Louis, MO), respectively. Phosphorylated Akt (pAkt) was assayed for PI3K activation and phosphorylated ERK (pERK) for MAPK activation.

### Protein extraction and Western blot analysis

Total proteins were extracted with RIPA buffer, to which protease inhibitors (complete Mini, GIBCO, Grand Island, NY) had been freshly added. Protein concentration was determined by the Bradford method (Bio-Rad, Richmond, CA). Protein extracts (20 μg) were separated by sodium dodecyl sulfate-polyacrylamide gel electrophoresis. Protein separation was performed in 10% polyacrylamide with 0.05% bis-acrylamide and transferred to a nitrocellulose membrane. Anti-PARP (1:1000 dilution; Cell Signaling Technology, Berverly, MA), anti-Bax (1:1000 dilution; Santa Cruz Biotechnology, Santa Cruz, CA), anti-pAkt (1:1000 dilution; Cell Signaling Technology, Berverly, MA), and anti-pERK (1:1000 dilution; Cell Signaling Technology, Berverly, MA) were used as primary antibodies. Anti-tubulin antibody (1:2000 dilution; NeoMarkers) was used for standardization of the protein amount. Immunoreactivity was detected by enhanced chemiluminescence (Supersignal, Thermo Scientific, Rockford, IL).

### Animal model

Transgenic TgN (SOD1G93A) 1GUR mice were obtained from the Jackson Laboratory (Bar Harbor, ME). These mice express high-copy numbers of the transgenic human mutant SOD1 containing the Gly 93 to Ala (G93A) substitution [51]. SOD1G93A transgenic mice were bred with B6SJLF1/J hybrid females. The presence of the human G93A transgene was confirmed by PCR assays of DNA in the tail tissue of transgenic offspring. Sixteen SOD1G93A transgenic mice and four wild (non-transgenic) mice were used for this experiment. All mice were housed under a 12-h light/dark cycle. All animal studies were carried out with the approval of the Institutional Animal Care and Use Committee (IACUC) of Seoul National University Hospital, which was accredited by the Association for the Assessment and Accreditation of Laboratory Animal Care International. Transgenic littermates were randomly assigned to a control (saline-treated) or GH-treated group. Intraperitoneal (i.p.) injections of 1.6749 μg (5 I.U.)/30 g GH (n = 9; 1 male and 8 females) or saline only (n = 7; 6 males and 1 female) were administrated weekly from postnatal week 11 to death. Body weight was recorded once a week until the mice were able to perform the behavioral task [22].

In ALS model mice, shaking of the limbs and suspension of the tail in the air are the initial symptoms of disease onset. At this stage, clonus, hyperreflexia, and crossed spread of spinal reflexes are also detectable in most mice. Subsequently, these mice develop gait impairments and paralysis of one (or both) hind limbs; in the final stage these mice are completely paralyzed. The end point was noted when ALS model mice could not right themselves within 30 s when placed on their sides on a flat surface; at this point they were sacrificed by cervical dislocation [52,53].

### Rotarod test

Using a previously described protocol, the Rotarod (Ugo Basile, Comerio, Italy) test was used in SOD1G93A mice from 11 weeks of age [54]. Training was performed from 10 weeks of age over 3 consecutive days, and testing began by placing mice individually on a rod (diameter = 4 cm), rotating at a constant speed of 16 rpm. Time taken before mice fell off the rod (3 min maximum) was recorded, and used a measure of motor function. During testing, three trials were performed, and the single longest duration spent on the rod was recorded. Mice were tested once a week until they were able to perform the task [22].

### Immunohistochemistry

For immunohistochemistry, mice were deeply anesthetized and perfused transcardially with cold 0.1 M PBS, followed by cold 4% paraformaldehyde for 10 min. Spinal cords were carefully dissected to obtain 4–10 mm long segments. Lumbar and cervical cords were identified by enlargement of the spinal cord. Tissues were postfixed in 4% paraformaldehyde for 8 h, and the tissue blocks produced were cryoprotected in a series of cold sucrose solutions of increasing concentration for 24 h. Transverse sections (30-μm thicknesses) were taken through lumbar and cervical cords. Cresyl violet acetate (Sigma, MO) staining was performed using these sections and incubated using the free-floating method (24–72 h, 4°C) with one of the following primary antibodies: monoclonal anti-glial fibrillary acidic protein, GFAP (1:500 dilutions; Sigma); or Bcl-2 (1:500 dilution; Santa Cruz Biotech, CA).

### Data analysis

The samples were analyzed in triplicate. For *in vitro* studies, values were expressed as mean ± SD, inter-group analysis by treatment was performed using Kruskal-Wallis or Mann-Whitney tests. Dose-dependent decrease in viability was determined using LD50. For *in vivo* studies, values are expressed as mean ± SD. Kaplan-Meier survival analysis and the Log-rank test were used for comparisons of survival. Body weight and rotarod test analysis by treatment were performed using the Mann-Whitney test. Statistical significance was accepted when p-value was less than 0.05. SPSS10.0 (SPSS Inc., Chicago, IL) program was used for statistical analysis.

## Abbreviations

ALS: Amyotrophic lateral sclerosis
fALS: Familial ALS
GH: Growth hormone
GSNO: S-nitrosoglutathione
HC: Homocysteine
hGH: Highly purified human growth hormone
MAPK: Mitogen Activated Protein Kinase
MTT: 3-[4,5-dimethylthiazol-2-yl]-2,5-diphenyltertazolium bromide
NO: Nitric oxide
PBS: Buffered saline
PI3K: Phosphatidylinositol 3-kinase
ROS: Reactive oxygen species
SOD1: Superoxide dismutase
W13: Wortmannin
